# Tomm34 is commonly expressed in epithelial ovarian cancer and associates with tumour type and high FIGO stage

**DOI:** 10.1186/s13048-019-0498-0

**Published:** 2019-03-27

**Authors:** Petr Muller, Philip J. Coates, Rudolf Nenutil, Filip Trcka, Roman Hrstka, Josef Chovanec, Veronika Brychtova, Borivoj Vojtesek

**Affiliations:** Masaryk Memorial Cancer Institute, Zluty kopec 7, 656 53 Brno, Czech Republic

**Keywords:** Tomm34, Chaperone, Epithelial ovarian cancer, Tumour, Immunohistochemistry, Ovary, Heat shock protein

## Abstract

**Background:**

Increased activity of the chaperones Hsp70 and Hsp90 is a common feature of solid tumours. Translocase of the outer mitochondrial membrane 34 (Tomm34) is a cochaperone of both Hsp70 and Hsp90 that was found to be overexpressed in colorectal, hepatocellular, lung and breast carcinomas. The expression profile of Tomm34 in ovarian cancer has not been investigated. Therefore, the aim of the current study was to investigate the expression pattern of Tomm34 in ovarian carcinomas and analyse its correlation with clinico-pathological parameters.

**Results:**

Epithelial ovarian cancers (140) were histologically classified based on their morphology and graded into two types comprising 5 histologic subgroups. Type I carcinomas comprise low grade serous (LGSC), clear cell (CCOC) and endometrioid (ENOC), type II comprises high grade serous carcinomas (HGSC) and solid, pseudoendometrioid, transitional carcinomas (SET). Tomm34 was more highly expressed in type II than type I carcinomas (*p* < 0.0001). Comparing tumours based on the mutation in the *TP53* gene revealed similar results, where mutant tumours exhibited significantly higher levels of Tomm34 (*p* < 0.0001). The decreased levels of Tomm34 in type I carcinomas were particularly evident in clear cell and mucinous carcinomas. The expression of Tomm34 was also positively correlated with FIGO stage (*r* = 0.23; *p* = 0.007). Tomm34 levels also indicated poor prognosis for patients with mutant p53.

**Conclusions:**

Our data indicate that Tomm34 is commonly expressed at high levels in epithelial ovarian cancers, except for the clear cell and mucinous subtypes. The expression of Tomm34 corresponds with the dualistic model of ovarian cancer pathogenesis where high grade, type II tumours exhibit higher expression of Tomm34 in contrast to type I tumours. These data are also comparable to the previous findings that Tomm34 is a marker of progression and poor prognosis in human cancer.

**Electronic supplementary material:**

The online version of this article (10.1186/s13048-019-0498-0) contains supplementary material, which is available to authorized users.

## Background

Epithelial ovarian cancer accounts for approximately 90% of ovarian tumours and is usually diagnosed only at advanced stages of the International Federation of Gynecology and Obstetrics (FIGO) system, accounting for its high mortality rate [1, 2]. Epithelial ovarian cancer is not a single disease and a dualistic model has been proposed to describe morphological subtypes and cell of origin [2, 3]. Type I ovarian cancers are low grade and develop slowly (including endometrioid, clear cell, mucinous and low grade serous adenocarcinomas) whereas type II cancers are the most common form, representing high-grade serous adenocarcinomas. It is also recognized that high and low-grade serous adenocarcinomas originate from precursor lesions in the fallopian tube, whilst the other histological type I tumours arise from endometriosis, germ cells or transitional cells, with an important role for distinct genetic alterations influencing the tumour characteristics [4–6]. Most notably, mutations in the p53 tumour suppressor are an overwhelming characteristic of Type II high grade serous tumours [7, 8]. Whilst many low grade lesions can be treated with surgery alone, high grade epithelial ovarian cancer is a difficult-to-treat disease that requires surgery plus combination chemotherapy, and even then recurrence is common (70–80% within two years), although this is improving with current targeted therapies for subsets of patients [9].

Increased chaperone activities are a universal feature of cancer and anti-Hsp90 or anti-Hsp70 therapeutics are under investigation for the treatment of various cancer types [10], including ovarian cancer [11, 12]. Chaperone activities are dependent on their interactions with co-chaperones that provide either protein folding or protein degradation functions. The protein folding activity of chaperones is hyperactive in cancers due to enhanced interactions of phosphorylated Hsp90 with Hsp70/Hsp90-organising protein (HOP, also known as STIP1) and reduced interaction with C-terminal Hsp70 interacting protein (CHIP, also known as STUB1) [13].

Translocase of the outer mitochondrial membrane 34 (Tomm34) is an additional component of the cellular chaperone system involved in protein folding. As the name suggests, Tomm34 was initially identified as being involved in mitochondrial protein processing [14, 15]. Subsequent studies have shown that Tomm34 interacts with both Hsp70 and Hsp90 and modifies their protein folding activities [16–20]. In cancer, high levels of Tomm34 have been reported in bladder, colorectal and breast cancers compared to their normal tissue counterparts [21–26]. In these cancers, Tomm34 promotes colorectal cancer cell growth [21] and is a biomarker of poor outcome in early invasive breast cancer [22] and bladder cancer [26]. As a tumour-associated protein, Tomm34 peptide vaccination is under investigation as a therapeutic option for colorectal cancer, with significant Tomm34 cytotoxic T-lymphocyte (CTL) response observed [27–29].

Previous data have indicated that the ovary also expresses *TOMM34* mRNA [21] although its expression in ovarian cancer has not been reported. Here, we investigated the levels of Tomm34 in ovarian cancers of mixed subtypes using immunohistochemistry. The data were correlated with tumour type and clinicopathological variables and demonstrate that Tomm34 is expressed at high levels in type II carcinomas and correlates with high FIGO stage.

## Results

### Patient details

**Fig. 1:**
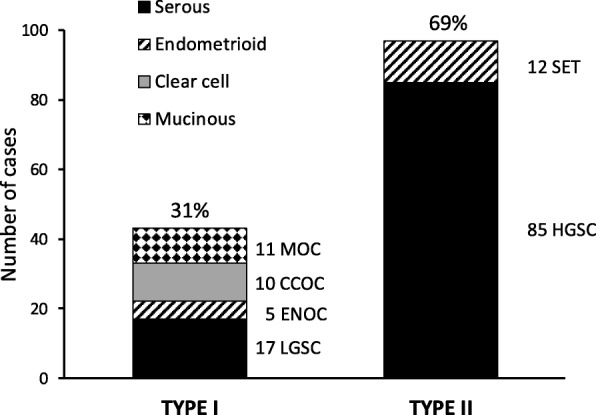
Distribution of histologic groups of 140 epithelial ovarian cancers. High grade tumours with endometrioid morphology and *TP53* mutation were classified as type II SET subgroup tumours

The patients (136) ranged in age from 29 to 86 years old (mean 59, median 59). Histological diagnosis classified tumours based on their morphology and grade into two types comprising 6 histologic subgroups. Type I carcinomas comprise low grade serous (LGSC), clear cell ovarian carcinoma (CCOC), endometrioid ovarian carcinoma (ENOC) and mucinous ovarian carcinoma (MOC). Type II include high grade serous carcinomas (HGSC) and solid, pseudoendometrioid, transitional carcinomas (SET). Since the carcinomas with serous and endometrioid morphology exhibited higher heterogeneity, the assignment of individual samples to histological subtypes was further verified by analysis of p53 status using sequencing and immunohistochemistry (Fig. 1). Serum CA125 levels varied from 6.6–42,415 U/ml, with 6 patients showing levels of less than 35. Thirty four (25%) cancers were FIGO stage 1; 15 (11%) FIGO 2; 65 (48%) FIGO 3 and 20 (15%) FIGO 4 (1 missing, *n* = 135). Fifty-one patients (38%) had residual disease (3 missing, *n* = 133) and 46 patients were alive and 90 were dead at last follow up. Twenty patients (14%) had a secondary tumour. Detailed information on individual cases is given in additional file 1.

### Tomm34 staining

**Fig. 2:**
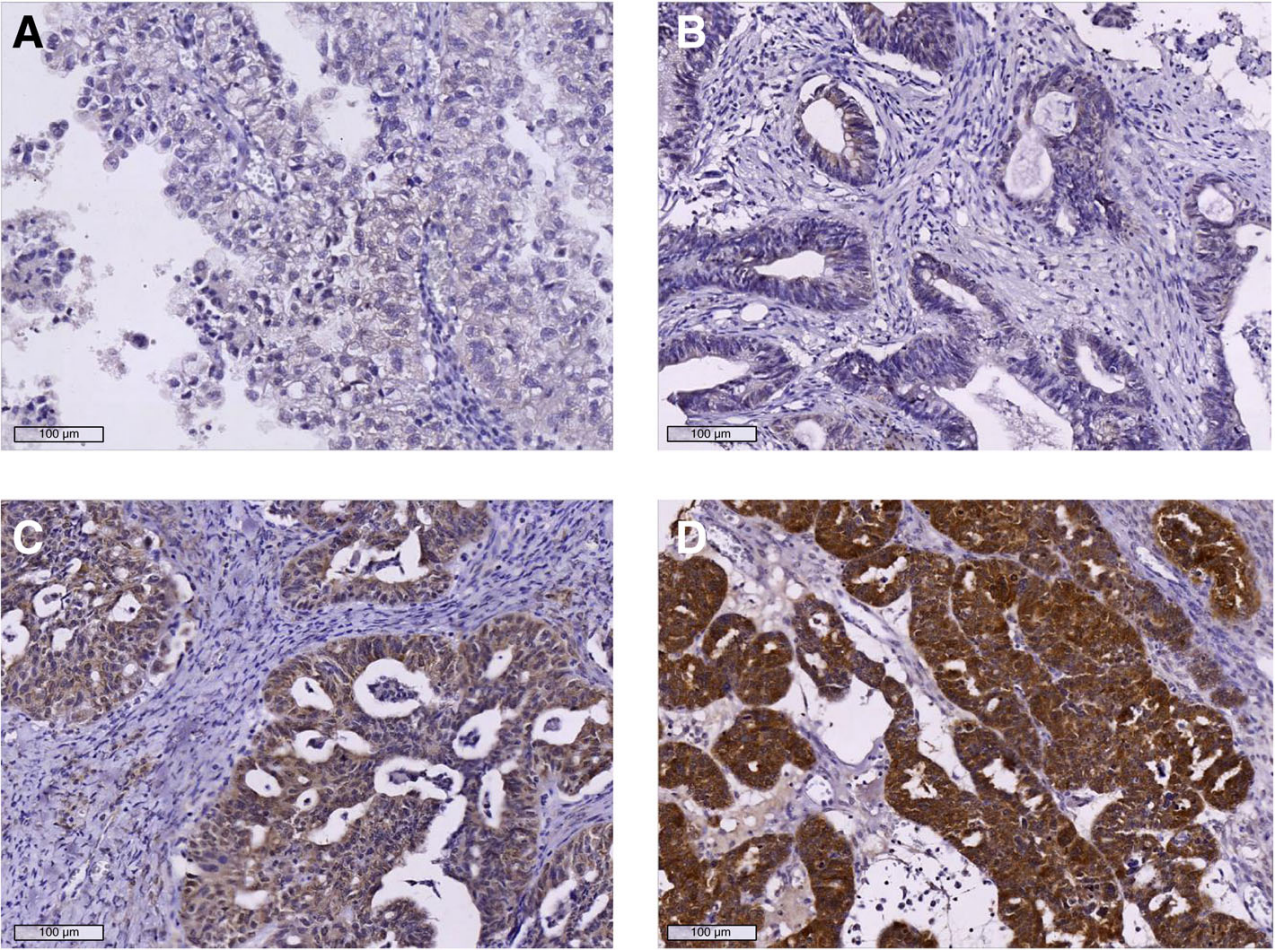
Representative staining patterns of Tomm34 in ovarian cancer. **a** histoscore 0, CCOC, grade 3. **b** histoscore 1, ENOC, grade 1. **c** histoscore 2, HGSC, grade 3. **d** histoscore 3, HGSC, grade 3. Magnification 100x

Tomm34 staining was seen in the cytoplasm of tumour cells. In the cohort of 136 ovarian cancers, Tomm34 was absent (score of 0) in 14 tumours, 35 cancers were scored as class 1; 44 as class 2; and 43 as class 3 (Fig. 2).

**Fig. 3:**
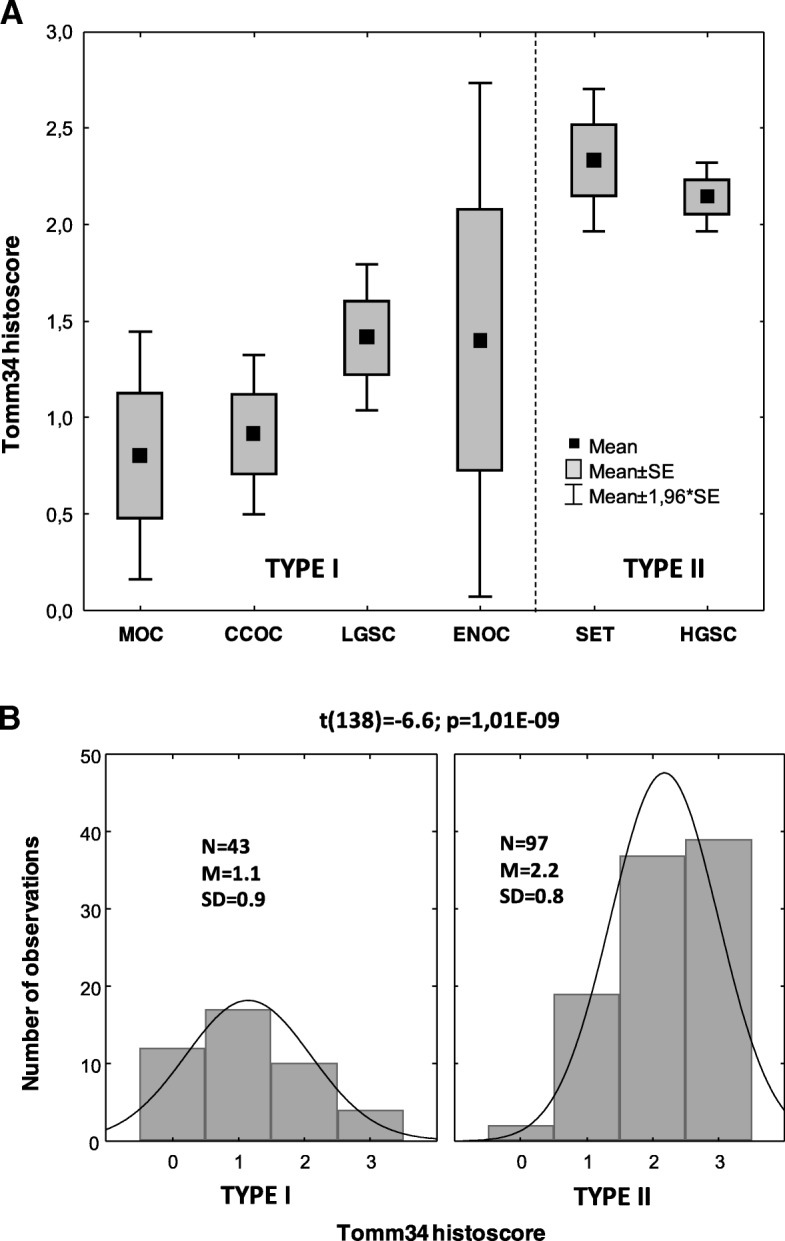
Expression of Tomm34 according to histological classification. **a** Distribution according to histological group. **b** The independent samples t-test revealed the most significant difference in Tomm34 between type I and type II tumours

We tested whether the level of Tomm34 corresponds with the histological type of tumour and whether it correlates with the dualistic model dividing the tumours into 2 types according to their pathogenesis. Figure 3a shows lower expression levels of Tomm34 in type I tumours (MOC, CCOC, LGSC and ENOC) compared to type II tumours (SET and HGSC). The lowest levels of Tomm34 were detected in MOC and CCOC (10 and 11 cases) when compared to the other samples t(138) = 5.6; *p* < 0.0001. The most statistically significant differences in Tomm34 were found when the cohort was classified according to tumours of types I and II t(138) = 6.6; *p* < 0.0001 (Fig. 3b). Comparing tumours bearing mutation in the *TP53* gene (94 cases) with *TP53* wild-type tumours (39 cases; 7 missing) revealed that *TP53* mutant tumours exhibited significantly higher levels of Tomm34 t(130) = 4.7; *p* < 0.0001.

**Fig. 4:**
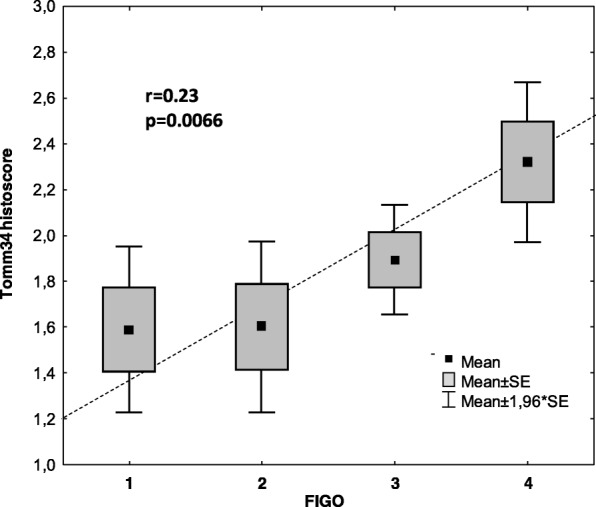
Relationship between FIGO stage and Tomm34 histoscore (0–3). Pearson correlation *r* = 0.23; *p* = 0.0066

Tomm34 staining also correlated with tumour progression represented by FIGO scale (Fig. 4). In accordance with this finding, higher values of Tomm34 were also found in patients who had residual disease after surgery (*n* = 51) versus the rest (*n* = 82) using independent samples t-test (*p* = 0.036). We also found that high Tomm34 levels correlate with pT status but not with presence of secondary tumour or response to therapy (Table 1). Analysis of patients with serous cancers as a separate group showed that Tomm34 staining correlated with pT status and high FIGO stage, but not with tumour grade or any of the other variables analysed (Table 2).

**Table 1:** Tomm34 correlates with tumour grade, FIGO stage and tumour type in ovarian cancer (*n* = 140)

Variable	Tomm34 0–1	Tomm34 2–3	*P* value
Tomm 34 score	50	90	
pT 1–2	29	22	
pT 3–4	19	57	0.0004
pN 0	19	18	
pN 1	11	16	0.454
M0	44	59	
M1	4	15	0.123
Secondary No	30	76	
Secondary Yes	8	11	0.456
Refractory	2	4	
Resistant	7	15	
Sensitive	29	63	0.966

pT size of primary tumour, pN degree of spread to regional lymph nodes given by histopathologic examination and M presence of distant metastasis. Tomm34 0–1 = IHC score of 0 or 1; Tomm34 2–3 = IHC score of 2 or 3; *P* value = Fisher exact 2-tailed test

**Table 2:** Tomm34 staining in serous ovarian cancers (*n* = 102)

Variable	Tomm 0–1	Tomm 2–3	*P* value
Tomm 34	29	73	
pT 1–2	15	16	
pT 3–4	12	49	0.0071
pN 0	8	13	
pN 1	7	14	1
M0	26	48	
M1	2	14	0.134
Secondary No	24	59	
Secondary Yes	4	11	1
Refractory	0	4	
Resistant	5	14	
Sensitive	19	48	0.707

pT size of primary tumour, pN degree of spread to regional lymph nodes given by histopathologic examination and M presence of distant metastasis. Tomm34 0–1 = IHC score of 0 or 1; Tomm34 2–3 = IHC score of 2 or 3; *P* value = Fisher exact 2-tailed test

To investigate the effects of Tomm34 on patient survival in more detail, we also analysed publicly available data using Kaplan-Meier Plotter for Ovarian Cancer (http://kmplot.com/analysis/index.php?p=service&cancer=ovar). These analyses indicated that *Tomm34* mRNA levels associate with poor overall survival (*p* = 0.036; HR = 1.29 (1.02–1.64; *n* = 506), poor progression-free survival (*p* = 0.0026; HR = 1.42 (1.13–1.8); *n* = 483) and poor post-progression free survival (*p* = 0.012; HR = 1.4 (1.07–1.82); *n* = 325) in patients with mutant p53 cancers (Additional file 2).

## Discussion

Cancer cells require a high level of protein synthesis due to their rapid proliferation and exhibit a high degree of stress due to genomic instability and lack of both oxygen and essential nutrients. To overcome such difficulties, cancer cells show up-regulation of many stress-induced proteins, including components of the protein chaperone system, particularly Hsp70 and Hsp90 [30–32]. Chaperone inhibition is therefore a clinically promising area [10], including for the treatment of ovarian cancer [11, 12]. Metabolic abnormalities are also a common finding in human cancers and the high rate of glucose uptake by tumour cells is utilised clinically for cancer imaging by 18F-2-deoxyglucose uptake and accumulation. Although it was originally thought that aerobic glycolysis in cancer cells (the Warburg effect) represented a lack of mitochondrial function, it is now clear that altered mitochondrial function represents a redeployment of glycolytic nutrients from catabolism to anabolism, required to meet the biosynthetic requirements of rapidly proliferating cancer cells, and NADPH production to help maintain redox balance [33–36].

These alterations to mitochondrial function relate to altered chaperoning of mitochondrial proteins [37]. Thus, targeting abnormal mitochondrial function and chaperoning of mitochondrial proteins is also a promising strategy for cancer therapeutics [38]. Hsp90 and Hsp70 play critical roles in mitochondrial protein stabilisation and folding [39] and various co-chaperones such as p23, Hsp40 and HOP are also involved [40–42]. In this respect, Tomm34 was originally identified as a potential mitochondrial import protein [15] and Tomm34 antibodies inhibit transport of preproteins into the mitochondria, whilst expression stimulates mitochondrial preprotein maturation and *TOMM34* siRNA inhibits this process [14]. Tomm34 is also reported to be increased as a component of compensatory adaptations to maintain normal rates of protein import in response to mitochondrial abnormalities [43]. On the other hand, and in agreement with our immunostaining data, Tomm34 exists predominantly in the cytoplasm rather than in mitochondria, suggesting it is involved in the transport of mitochondrial preproteins in an unfolded state prior to import [14, 17, 44].

Our data indicate that Tomm34 is commonly expressed at high levels in human ovarian cancers, except for the MOC and CCOC subtype, where high level Tomm34 is rarely seen. Within the different sub-types of ovarian cancer, high levels of Tomm34 associate with higher stage and higher grade cancers and similar findings are seen within the sub-type of serous cancers. Most notably, we have found that Tomm34 associates with poor survival of patients with p53-mutant ovarian cancers. These data are also comparable to the findings that Tomm34 is a marker of poor outcome and a predictor of distant metastasis in breast cancer [22, 23]. Thus, as with breast cancer patients, Tomm34 may serve as part of a panel of markers for prognostic determination in ovarian cancers. In this respect, proteomic analysis of an animal model of ovarian cancer revealed up-regulation of numerous proteins involved in metabolic processes, including endoplasmic stress responses, mitochondrial systems and chaperones such as Hsp70 and Grp78 [45].

The mechanism for high level expression of Tomm34 is unclear from our studies. Data from the COSMIC database (http://cancer.sanger.ac.uk/cosmic) indicate that only 1.5% of 729 ovarian cancers tested show copy number variation gain of *TOMM34*, indicating that gene amplification is a rare event. However, Tomm34 is transcriptionally regulated by NRF-1 and NRF-2 [46, 47] and these latter are implicated in directing metabolic reprogramming during stress [48] and are often hyperactive in cancers. Although further research will be required to elucidate the mechanisms that regulate Tomm34 in ovarian and other cancers, our data imply that Tomm34 has pro-tumourigenic actions and may serve as a useful prognostic indicator and potential therapeutic target in ovarian cancers.

## Conclusions

The co-chaperone Tomm34 is frequently expressed in epithelial ovarian cancers. Immunohistochemical identification of Tomm34 may be a useful adjunct to provide prognostic information and may serve as an immunogenic target for therapy.

## Methods

### Ovarian cancer samples and TMA construction

A total of 140 samples of ovarian cancer with anonymised clinicopathological and survival data were available for immunohistochemical staining of Tomm34. All tissues had been removed during surgery, fixed in formalin and processed into paraffin wax for histopathological diagnosis. Tissue microarrays were constructed using five separate cores (1.5 mm diameter each) selected from different regions of each tumour. Clinicopathological information includes age, histological subtype, p53 status, TNM status, grade, FIGO stage, CA125 levels, tumour response to therapy, progression free survival and overall survival. Histological classification was performed as follows: type I carcinomas comprise low grade serous carcinoma (LGSC), clear cell ovarian carcinoma (CCOC), endometrioid ovarian carcinoma (ENOC) and mucinous ovarian carcinoma (MOC). Type II include high grade serous carcinomas (HGSC) and solid, pseudoendometrioid, transitional carcinomas (SET). The study was approved by the MMCI biobank and all patients gave informed consent for the use of their tissues for research.

### Tomm34 antibody generation and characterization

Full-length recombinant Tomm34 purified from *E. coli* was used for immunization of mice. The hybridomas producing monoclonal antibodies were generated by Moravian Biotechnology. The clones were characterized by immunoblotting and immunohistochemistry. Hybridoma clone Tomm34–4.1 was used due to its highest sensitivity and specificity in immunohistochemistry. Specificity of the antibody was tested on MCF7 Tomm34 −/− cell line, where the Tomm34 gene was removed by CRISPR knock-out (Additional file 3).

### Tissue microarrays, immunohistochemistry and scoring

Tissue blocks of primary tumours were fixed in 4% neutral formaldehyde for approximately 24 h before processing into paraffin wax. Representative cores of tumour were selected from the diagnostic blocks by an experienced histopathologist. The tissue microarrays comprised 45 tissue cores consisting of nine tumour samples each represented by five cores from the original paraffin block. Endogenous peroxidase was blocked with 3% hydrogen peroxide in phosphate buffered saline (PBS), pH 7.5, for 15 min. Antigen retrieval was performed in 1 mM EDTA–NaOH (pH 8.0) for 40 min at 93 °C. Primary antibodies were applied overnight at 4 °C at 2 μg/ml in antibody diluent (DakoCytomation, Denmark). The antibody was visualized using peroxidase labelled polymer conjugated to goat anti-mouse immunoglobulins and EnVision+ System containing DAB chromogen (DakoCytomation, Denmark). The nuclei were counterstained with Gill’s haematoxylin before permanent mounting. Neoplastic cells were classified into four staining intensities: 0 no staining, 1 weak staining, 2 moderate staining, 3 strong staining.

### Statistical analyses

The independent samples t-test was used to compare two sets of independent of and identically distributed samples; *P* values < 0.05 were considered significant. Relationships between two continuous variables were tested by Pearson correlation where correlation coefficient r was considered significant if *P* values < 0.05.

## Additional files

Additional file 1: Clinicopathological data of individual cases (PDF 276 kb)

Additional file 2: Survival analysis based on Tomm34 expression in epithelial ovarian cancers with p53 mutation. (A) Overall survival, (B) progression free survival and (C) post progression survival. (PDF 204 kb)

Additional file 3: Detection of Tomm34 by mouse monoclonal antibody Tomm34.4.1. MCF7 cells with knockout expression of Tomm34 gene were transfected with constructs encoding HA-tag labelled full-length Tomm34 protein and its TPR1 (aa 1–188) and TPR2 (188–309) domains. Cell lysates were separated by SDS-PAGE, blotted and the membranes were probed with either anti-HA tag antibody or Tomm34.4.1 monoclonal antibody. Protein loading was tested by probing the membrane with anti-Actin antibody. (PDF 158 kb)
