# Supplementary material for: Tomm34 is commonly expressed in epithelial ovarian cancer and associates with tumour type and high FIGO stage
Source: J Ovarian Res. 2019 Mar 27;12:30. doi: 10.1186/s13048-019-0498-0 (PMC6436220; doi:10.1186/s13048-019-0498-0)
Supplement: Supplementary file 1 — Clinicopathological data of individual cases (PDF 276 kb) [file 13048_2019_498_MOESM1_ESM.pdf]

Patient No.	Age at time of surgery	FIGO	CA125 value at time of surgery [U/ml]	Residual disease	Therapy response	Progression	Progression free survival [months]	Overall survival [months]	Dead	Histological subtype	Type	Grade	p53 mutation	Tomm34 histoscore
1	60	IV	1995,9	Yes	RESITANT	Yes	26	89	No	HGSC	2	3	MIS	1
2	55	IIB	201	No	SENSITIV	Yes	67	85	Yes	MOC	1	1	WT	1
3	56	IIIC	3206	No	SENSITIV	No	62	62	No	HGSC	2	3	DEL	2
4	41	IA	146,6	No		Yes	17	27	No	SET	2	2	MIS	3
5	64	IIIC	115,4	Yes	RESITANT	Yes	17	30	Yes	HGSC	2	3	NONSENSE	3
6	70	IV	2043,2	Yes	SENSITIV	No	120	120	No	HGSC	2	3	NONSENSE	2
7	39	IIIA	72,6	Yes	SENSITIV	Yes	22	47	Yes	HGSC	2	3	MIS	3
8	54	IA	53,1	No		Yes	57	74	No	MOC	1	1	WT	0
9	50	IIIC	5090,3	Yes	SENSITIV	No	80	80	No	HGSC	2	3	NONSENSE	2
10	58	IIIA	770,9	No		No	133	133	No	CCOC	1	3	WT	0
11	55	IIIC	4695,6	Yes	SENSITIV	Yes	17	29	Yes	HGSC	2	3	MIS	1
12	72	IV	625	Yes	SENSITIV			79	No	HGSC	2	3	MIS	3
13	65	IIIC	218	No		No	49	49	No	HGSC	2	3	WT	3
14	56	IA	465,5	No	REFRACT	No	71	71	No	HGSC	2	3		3
15	74	IA	82	No		Yes	86	109	No	MOC	1	2	WT	1
16	60	IIC	351,6	No	SENSITIV	No	103	103	No	HGSC	2	3	NONSENSE	2
17	36	IIIC	98	No	SENSITIV	Yes	19	44	Yes	LGSC	1	1	WT	1
18	53	IV	2483,7	Yes	RESITANT	No	69	69	No	HGSC	2	3	DEL	3
19	60	IV	2823,4	Yes	SENSITIV	Yes	74	74	Yes	HGSC	2	3	MIS	2
20	57	IV	20930,2	Yes	SENSITIV	No	123	123	No	HGSC	2	3	MIS	1
21	64	IIIC	2611,9	Yes	SENSITIV	Yes	51	106	No	HGSC	2	3	DEL	2
22	45	IIIC	52	No	SENSITIV	Yes	18	38	Yes	HGSC	2	3	MIS	3
23	63	IIIC	133	Yes	SENSITIV	Yes	38	41	Yes	HGSC	2	3	NONSENSE	3
24	63	IIIB	163	Yes	REFRACT	Yes	3	3	Yes	MOC	1	1	WT	0
25	53	III C	132,66	No	SENSITIV	Yes	8	52	Yes	ENOC	2	2	WT	2
26	42	IIIC	3070,6	No	SENSITIV	Yes	36	112	Yes	LGSC	1	2	WT	2
27	67	IIIC	5887,5	Yes	SENSITIV	Yes	24	53	Yes	SET	2	3	MIS	3
28	65	IIC	440	No	SENSITIV	Yes	53	71	Yes	LGSC	1	1	WT	1
29	66	IB	1896,7	No	SENSITIV	No	122	122	No	MOC	1	3	MIS	2
30	46	IIIA	6794,4	No	SENSITIV	Yes	20	41	Yes	HGSC	2	3	NONSENSE	3
31	48	IA	43,2	No	SENSITIV	Yes	17	17	Yes	HGSC	2	3	MIS	2
32	79	IIIA	21,8	No	SENSITIV	Yes	18	18	Yes	SET	2	3	NONSENSE	2
33	53	IB	49	No	SENSITIV	No	49	49	No	LGSC	1	1	MIS	2
34	37	IIIC	150	No	SENSITIV	No	77	77	No	LGSC	1	2	WT	1
35		ND				No	75	75	No	LGSC	1	1		2
36	46	IV	3327,4	No	SENSITIV	Yes	10	23	Yes	HGSC	2	3	MIS	2
37	42	IIIC	2397,7	Yes	SENSITIV	No	65			HGSC	2	3	MIS	2
38	58	IIIC	259	Yes	RESITANT	No	105	105	No	LGSC	1	1		2
39	47	IIC	220,9	No		No	52	52	No	CCOC	1	3	SIL	0
40	59	IIIC			REFRACT	Yes	17	17	Yes	LGSC	1	1	WT	2
41		ND				Yes	9	40	Yes	HGSC	2	3		2
42	74	IA		No	SENSITIV	No	124	124	No	CCOC	1	1	WT	0
43	40	IIIC	1382	No	RESITANT	Yes	6	6	Yes	HGSC	2	3	MIS	1
44	59	IIIC	41	No	SENSITIV	Yes	1	1	Yes	HGSC	2	3	WT	1
45	61	IA	184,3	No	SENSITIV			106	No	HGSC	2	2	MIS	3
46	66	IIIC	4000	Yes	SENSITIV	Yes	2	2	Yes	HGSC	2	3	MIS	1
47	53	IV	2160	Yes	SENSITIV	No	64	64	No	HGSC	2	3	MIS	3

Patient No.	Age at time of surgery	FIGO	CA125 value at time of surgery [U/ml]	Residual disease	Therapy response	Progression	Progression free survival [months]	Overall survival [months]	Dead	Histological subtype	Type	Grade	p53 mutation	Tomm34 histoscore
48	68	IIIC	499,2	Yes	RESITANT	Yes	12	56	Yes	HGSC	2	3	DEL	3
49	56	IC	124	No	SENSITIV			57	No	HGSC	2	3	NONSENSE	3
50	62	IIIC	114	No						CCOC	1	2	WT	1
51	62	IV	1386	No	RESITANT	Yes	5	13	Yes	SET	2	3	MIS	3
52	59	IIIC	267	No	REFRACT	No	59	59	No	CCOC	1	2	MIS	1
53	64	IC	69	No	SENSITIV	Yes	16	30	Yes	HGSC	2	3	WT	3
54	63	IIIC	40	No	SENSITIV	Yes	42	57	No	HGSC	2	3	MIS	3
55	80	IB	24	No	SENSITIV	Yes	9	11	Yes	LGSC	1	1	MIS	1
56	54	IC		No	SENSITIV	Yes	30	82	No	ENOC	1	1	WT	0
57	43	IIIC	1481	No	SENSITIV	Yes	7	51	No	HGSC	2	3	MIS	3
58	53	IIIC		Yes	SENSITIV	Yes	5	5	Yes	HGSC	2	3	NONSENSE	2
59	67	IC	261	No	RESITANT	Yes	27	32	Yes	LGSC	1	1	WT	1
60	49	IC	17,7	No	SENSITIV	No	83	83	No	ENOC	1	1	WT	3
61	70	IIC	197,1		SENSITIV	Yes	45	60	Yes	LGSC	1	2	MIS	2
62	59	IC	242	No		Yes	19			HGSC	2	3	WT	0
63	53	IIIC	4528	Yes	SENSITIV	Yes	11	23	Yes	HGSC	2	3	WT	3
64	58	IV	427,5	Yes	RESITANT	Yes	45	90	No	HGSC	2	3	MIS	3
65	70	IIIC	42415,1	No		Yes	24	66	Yes	HGSC	2	3	MIS	2
66	59	IA	276,1	No	SENSITIV	Yes	16	42	Yes	CCOC	1	3	WT	2
67	53	IIIB	458,4	No	SENSITIV	Yes	9	17	Yes	LGSC	1	1	NONSENSE	0
68	60	IV	216,7	Yes	RESITANT	No	134	134	No	MOC	1	3	MIS	0
69	62	IIIC	199,6	Yes	RESITANT	Yes	6	17	Yes	CCOC	1	3	WT	1
70	59	IIC	677,5	No	SENSITIV	Yes	32	85	No	HGSC	2	3	NONSENSE	2
71	29	IIIC	313,6	No	SENSITIV	Yes	2	2	Yes	HGSC	2	3	MIS	3
72	70	IIB	1019,1	No		Yes	27	72	Yes	HGSC	2	3	DEL	1
73	56	IA	290	No	SENSITIV	Yes	10	27	Yes	HGSC	2	2	DEL	1
74	59	IIIC	588,9	No	SENSITIV	No	60	60	No	HGSC	2	3	NONSENSE	3
75		IIIC				Yes	17	22	Yes	HGSC	2	2		2
76	78	IIIB	341,6	Yes	RESITANT	Yes	33	111	Yes	HGSC	2	2	MIS	3
77	76	IIC	113,3	Yes	SENSITIV	No	73	73	No	HGSC	2	3	MIS	1
78	58	III-IV	9734	Yes		Yes	12	12	Yes	HGSC	2	3	MIS	3
79	63	IIIC	2225	No	SENSITIV	Yes	42	113	No	HGSC	2	3	MIS	1
80	53	IIIC	101,7	No	SENSITIV	Yes	42	42	Yes	SET	2	3	MIS	2
81	62	IA	4276,9	No		No	115	115	No	LGSC	1	1	WT	3
82	49	IIIC	2964,2	Yes	SENSITIV	Yes	43	60	No	SET	2	3	DEL	2
83	70	IIIC	500,4	Yes	SENSITIV	Yes	7	25	Yes	HGSC	2	3	NONSENSE	2
84	55	IIIC	1394,3	No	RESITANT	Yes	28	58	Yes	HGSC	2	3	MIS	2
85	52	IIIC	113,1	Yes	SENSITIV	Yes	47	59	No	SET	2	3	MIS	1
86	52	IIIC	219,5	No		No	114	114	No	LGSC	1	2	WT	1
87	65	IC	1094,2	No	SENSITIV	Yes	22	24	Yes	HGSC	2	3	MIS	2
88	66	IA	54,5	No	SENSITIV	Yes	7	9	Yes	CCOC	1	3	WT	1
89	62	IIIC	1000	Yes	SENSITIV	Yes	11	19	Yes	CCOC	1	3	WT	1
90	81	IA	146,5	No	SENSITIV	Yes	11	22	Yes	MOC	1	1		0
91	76	IV	19690,3	Yes	SENSITIV	Yes	27	81	No	HGSC	2	2	NONSENSE	2
92	73	IIIC	277,9	No	SENSITIV	Yes	56	56	Yes	HGSC	2	3	NONSENSE	3
93	66	IC	139,1	No	SENSITIV	Yes	11	66	Yes	LGSC	1	1		1
94	55	III-IV	1327,2	No	SENSITIV	Yes	11	20	Yes	ENOC	1	2	WT	3

Patient No.	Age at time of surgery	FIGO	CA125 value at time of surgery [U/ml]	Residual disease	Therapy response	Progression	Progression free survival [months]	Overall survival [months]	Dead	Histological subtype	Type	Grade	p53 mutation	Tomm34 histoscore
95	72	IIIC	885	No	SENSITIV	No	90	90	No	HGSC	2	3	MIS	1
96	69	IV	3550,2	Yes	SENSITIV	Yes	6	34	Yes	HGSC	2	3	MIS	3
97	48	IC	60,4	No	SENSITIV	Yes	14	28	Yes	CCOC	1	3	WT	2
98	70	IIIC	261,6	Yes	SENSITIV	No	63	63	No	HGSC	2	2	MIS	3
99	53	IIIC	7293,8	Yes	RESITANT	Yes	11	11	Yes	HGSC	2	3	MIS	3
100	64	IC	248,8	No	SENSITIV	Yes	12	14	Yes	HGSC	2	3	MIS	1
101	66	IB	181,3	No	SENSITIV	Yes	17	38	Yes	HGSC	2	2	MIS	1
102	63	IIIB	140	No	SENSITIV	Yes	34	121	No	MOC	1	1	WT	1
103	54	IIC	1124,1	No	SENSITIV	No	47	47	No	HGSC	2	3	DEL	2
104	50	ND	10537	No	SENSITIV	Yes	77	118	Yes	HGSC	2	3	MIS	1
105	49	IV	2203,4	Yes	SENSITIV	Yes	20	62	Yes	HGSC	2	3	NONSENSE	3
106	86	IB	1474,6	No		Yes	15	30	Yes	LGSC	1	1	WT	1
107	46	IIIC	1443,7	Yes	SENSITIV	Yes	34	64	No	HGSC	2	2	DEL	3
108	67	IC	225,8	Yes	SENSITIV	Yes	18	56	Yes	LGSC	1	1	WT	2
109	42	IIIC	259,6	Yes	SENSITIV	No	101	101	No	MOC	1	3	WT	0
110	61	IC	238,8	No		Yes	46	49	No	HGSC	2	3	NONSENSE	1
111	62	IIIC	1562,8	Yes	RESITANT	Yes	34	75	No	HGSC	2	3	MIS	2
112	59	IA	81,7	No	SENSITIV	No	124	124	No	HGSC	2	3	MIS	3
113	54	IIIA	7750	Yes	RESITANT	Yes	26	35	Yes	LGSC	1	1	WT	1
114	52	IC		No	SENSITIV	Yes	33	71	No	SET	2	3	MIS	2
115	85	IIIC	1928	No	RESITANT	Yes	15	56	Yes	HGSC	2	2		2
116	62	IB	884,6	No	SENSITIV	No	49	49	No	HGSC	2	3	MIS	2
117	76	IIIC	141,9	No	SENSITIV	Yes	15	39	Yes	SET	2	3	MIS	3
118	67	IV	274,2	Yes	RESITANT	Yes	17	31	Yes	HGSC	2	3	MIS	2
119	56	IIA	243,5	Yes	REFRACT	Yes	52	52	Yes	HGSC	2	3	DEL	2
120	57	IIIC	992,1	No	SENSITIV	Yes	1	1	Yes	HGSC	2	3	MIS	2
121	55	IV	1170,6	Yes	SENSITIV	No	52	52	No	HGSC	2	2	DEL	3
122	54	IIIC	2906,8	No	SENSITIV	Yes	7	18	Yes	LGSC	1	1	WT	0
123	62	IV	457,4	Yes	RESITANT	Yes	32	66	No	HGSC	2	3	NONSENSE	2
124	48	IIC	72	Yes	SENSITIV	Yes	7	16	Yes	ENOC	1	1	WT	1
125	45	IA		No		Yes	4	25	Yes	CCOC	1	3	WT	1
126	50	IV	481,8		SENSITIV	Yes	15	15	Yes	HGSC	2	3	MIS	2
127	49	IIC	345,1	No	SENSITIV	Yes	19	58	No	HGSC	2	3	DEL	2
128	50	IV	9046,4	No	REFRACT	Yes	19	28	Yes	HGSC	2	3	NONSENSE	2
129	77	IIC	23,9	Yes	SENSITIV	Yes	17	37	Yes	HGSC	2	3	MIS	3
130	50	IA	6,6	No		Yes	71	71	Yes	ENOC	1	1	WT	0
131	48	IIC		No	SENSITIV			102	No	HGSC	2	3	MIS	2
132	51	IIB	1510,7	No	SENSITIV	Yes	20	54	Yes	SET	2	3	DEL	2
133	46	IC	15,3	No	SENSITIV	No	125	125	No	SET	2	3	NONSENSE	3
134	63	IV	128,4	No	SENSITIV	Yes	18	96	No	HGSC	2	3	MIS	2
135	68	IIIA	353,4	Yes	RESITANT	No	109	109	No	HGSC	2	3	MIS	0
136	76	IIIC	1508,9	Yes	RESITANT	Yes	7	15	Yes	HGSC	2	2	MIS	3
137	65	IIIC	236,8	Yes	RESITANT	No	98	98	No	HGSC	2	3	NONSENSE	3
138	53	IV	174,5	No	SENSITIV					MOC	1	1	MIS	3
139	49	IIIC	7465	Yes	SENSITIV					HGSC	2	3	WT	3
140	59	IIB	1626,5	Yes	SENSITIV					HGSC	2	2	MIS	1
