# Supplementary material for: Tomm34 is commonly expressed in epithelial ovarian cancer and associates with tumour type and high FIGO stage
Source: J Ovarian Res. 2019 Mar 27;12:30. doi: 10.1186/s13048-019-0498-0 (PMC6436220; doi:10.1186/s13048-019-0498-0)
Supplement: Supplementary file 2 — Survival analysis based on Tomm34 expression in epithelial ovarian cancers with p53 mutation. (A) Overall survival, (B) progression free survival and (C) post progression survival. (PDF 204 kb) [file 13048_2019_498_MOESM2_ESM.pdf]

A

Overall survival OS

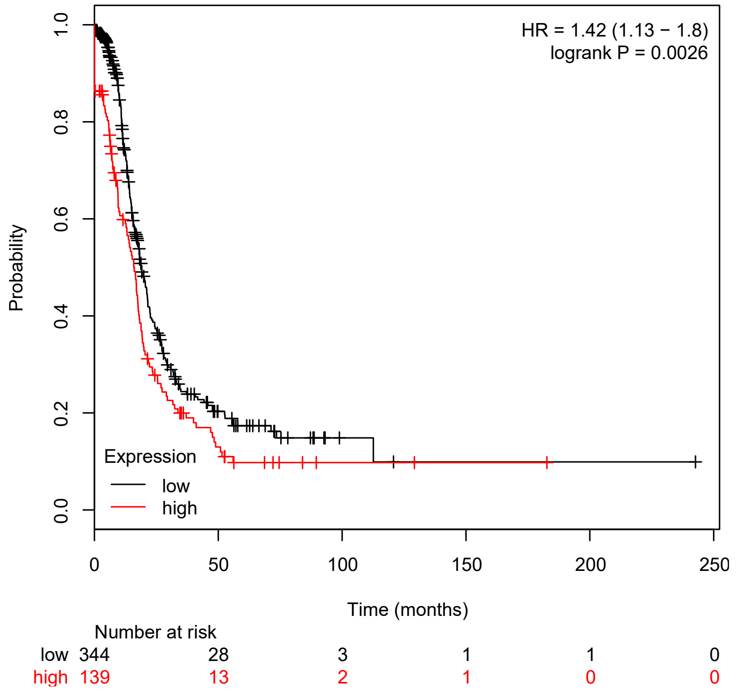

Affymetrix ID: 201870_at TOMM34
Survival: Overall survival OS
Number of cases: 506
Cutoff value used in analysis: 1041
Expression range of the probe: 243 - 3252
TP53 mutation: mutated
P value: 0.0363
FDR: over 50%

B

Progression free survival PFS

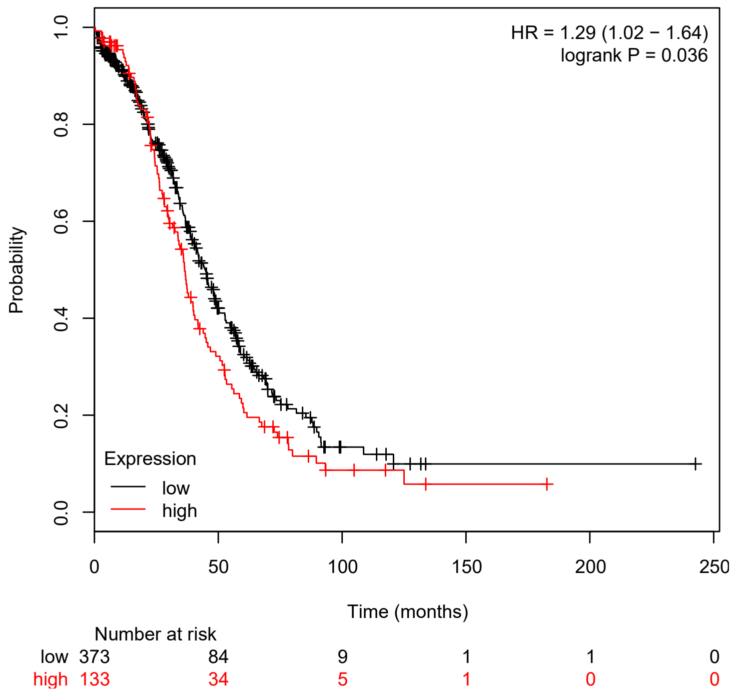

Affymetrix ID: 201870_at TOMM34
Survival: Progression free survival PFS
Number of cases: 483
Cutoff value used in analysis: 1026
Expression range of the probe: 243 - 3252
TP53 mutation: mutated
P value: 0.0026
FDR: over 50%

C

Post progression survival PPS

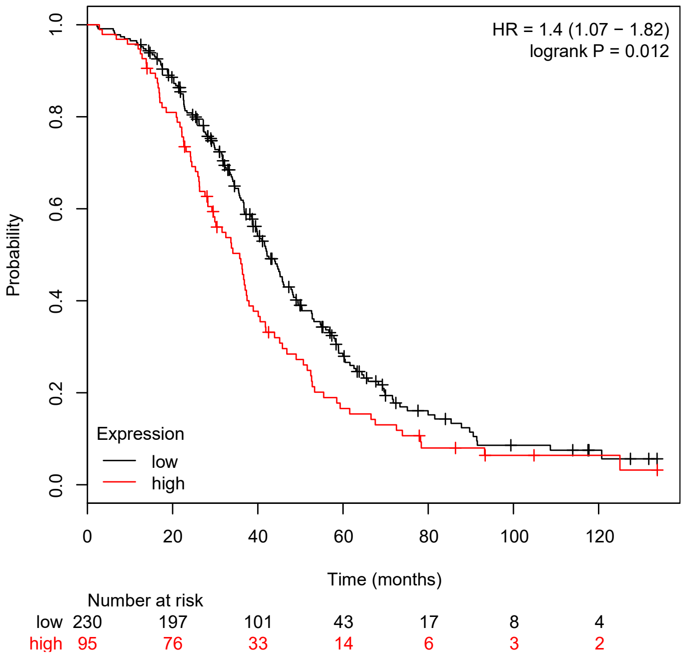

Affymetrix ID: 201870_at TOMM34
Survival: Post progression survival PPS
Number of samples: 325
Cutoff value used in analysis: 1094
Expression range of the probe: 244 - 3252
TP53 mutation: mutated
P value: 0.0121
FDR: over 50%
