# Supplementary material for: Tomm34 is commonly expressed in epithelial ovarian cancer and associates with tumour type and high FIGO stage
Source: J Ovarian Res. 2019 Mar 27;12:30. doi: 10.1186/s13048-019-0498-0 (PMC6436220; doi:10.1186/s13048-019-0498-0)
Supplement: Supplementary file 3 — Detection of Tomm34 by mouse monoclonal antibody Tomm34.4.1. MCF7 cells with knockout expression of Tomm34 gene were transfected with constructs encoding HA-tag labelled full-length Tomm34 protein and its TPR1 (aa 1–188) and TPR2 (188–309) domains. Cell lysates were separated by SDS-PAGE, blotted and the membranes were probed with either anti-HA tag antibody or Tomm34.4.1 monoclonal antibody. Protein loading was tested by probing the membrane with anti-Actin antibody. (PDF 158 kb) [file 13048_2019_498_MOESM3_ESM.pdf]

Additional file 3

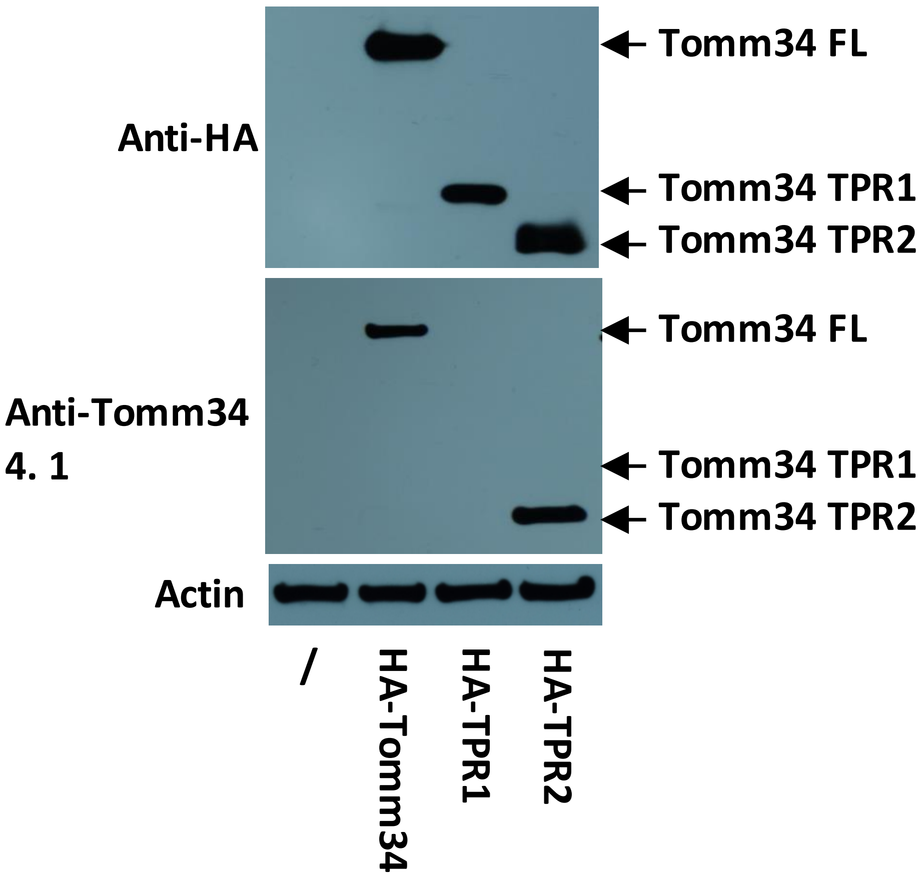

Characterization of Tomm34.4.1 antibody. MCF7 cells with knockout expression of *Tomm34* gene were transfected with constructs encoding HA-tag labeled full-length Tomm34 protein and its TPR1 (aa 1-188) and TPR2 (188-309) domains. Next, non-transfected and transfected cell lysates were separated by SDS-PAGE, blotted and the membranes were probed with either anti-HA tag antibody or anti-Tomm34 4. 1 monoclonal antibody. Protein loading was tested by probing the membrane with anti-Actin antibody.
